# Histone Methyltransferases G9a/*Ehmt2* and GLP/*Ehmt1* Are Associated with Cell Viability and Poorer Prognosis in Neuroblastoma and Ewing Sarcoma

**DOI:** 10.3390/ijms242015242

**Published:** 2023-10-17

**Authors:** Barbara Kunzler Souza, Natalia Hogetop Freire, Thiago Santos Monteiro, Alice Laschuk Herlinger, Mariane Jaeger, Matheus G. S. Dalmolin, Caroline Brunetto de Farias, Lauro Gregianin, André T. Brunetto, Algemir L. Brunetto, Carol J. Thiele, Rafael Roesler

**Affiliations:** 1 Cancer and Neurobiology Laboratory, Experimental Research Center, Clinical Hospital (CPE-HCPA), Federal University of Rio Grande do Sul, Porto Alegre 90035-003, Brazilandrebrunetto@ici.ong (A.T.B.);; 2 National Science and Technology Institute for Children’s Cancer Biology and Pediatric Oncology—INCT BioOncoPed, Porto Alegre 90035-003, Brazil; 3 Epigenica Biosciences, Canoas 92035-000, Brazil; tsmonteiro@gmail.com; 4 Children’s Cancer Institute, Porto Alegre 90620-110, Brazil; 5 Department of Pediatrics, School of Medicine, Federal University of Rio Grande do Sul, Porto Alegre 90035-003, Brazil; 6 Pediatric Oncology Service, Clinical Hospital, Federal University of Rio Grande do Sul, Porto Alegre 90035-003, Brazil; 7 Pediatric Oncology Branch, Center for Cancer Research, National Cancer Institute, National Institutes of Health, Bethesda, MD 20892, USA; 8 Department of Pharmacology, Institute for Basic Health Sciences, Federal University of Rio Grande do Sul, Porto Alegre 90035-003, Brazil

**Keywords:** G9a, GLP, histone methyltransferase, neuroblastoma, Ewing sarcoma, pediatric cancer

## Abstract

Changes in epigenetic programming have been proposed as being key events in the initiation and progression of childhood cancers. HMT euchromatic histone lysine methyltransferase 2 (G9a, EHMT2), which is encoded by the *G9a* (*Ehmt2*) gene, as well as its related protein GLP, which is encoded by the *GLP*/*Ehmt1* gene, participate in epigenetic regulation by contributing to a transcriptionally repressed chromatin state. G9a/GLP activation has been reported in several cancer types. Herein, we evaluated the role of G9a in two solid pediatric tumors: neuroblastoma (NB) and Ewing sarcoma (ES). Our results show that *G9a/Ehmt2* and *GLP*/*Ehmt1* expression is higher in tumors with poorer prognosis, including St4 International Neuroblastoma Staging System (INSS) stage, *MYCN* amplified NB, and metastatic ES. Importantly, higher *G9a* and *GLP* levels were associated with shorter patient overall survival (OS) in both NB and ES. Moreover, pharmacological inhibition of G9a/GLP reduced cell viability in NB and ES cells. These findings suggest that G9a and GLP are associated with more aggressive NB and ES tumors and should be further investigated as being epigenetic targets in pediatric solid cancers.

## 1. Introduction

Histone methylation is a mechanism for generating epigenetic marks that either activate or repress transcription depending on the target gene. Histone methyltransferases (HMTs) mediate the addition of up to three methyl groups to a histone lysine residue and up to two groups to a histone arginine residue [1,2]. The HMT euchromatic histone lysine methyltransferase 2 (G9a, EHMT2), which is encoded by the *G9a* (*Ehmt2*) gene, and its related protein (GLP, EHMT1), which is encoded by the *GLP* (*Ehmt1*) gene, mediate mono- and dimethylation (H3K9me1 and H3K9me2) of H3 lysine 9 (H3K9), resulting in a condensed and transcriptionally repressed chromatin structure [3,4,5,6,7]. The abnormal activation of this process, in conjunction with G9a overexpression, has been observed in several adult solid cancer types [8,9,10]. G9a forms homomeric and heteromeric complexes with the G9a-related methyltransferase, G9a-like protein (EHMT1 or GLP), which is encoded by the *Ehmt1* gene, to catalyze both H3K9me1 and H3K9me2 [11,12,13].

Neuroblastoma (NB), which likely arises from developing neural crest cells, is the most common extra-cranial pediatric solid tumor in children. High-risk disease with poor prognosis is associated with *MYCN* amplification or unbalanced 11q. Currently, only about half of high-risk patients achieve long-term remission with multimodal therapy [14,15]. Ewing sarcoma (ES) is an aggressive pediatric tumor that occurs either in the bone or soft tissues. Moreover, ES likely originates from neural crest or mesenchymal stem cells and is the prototypical cancer type defined by a single specific genetic alteration, namely a chromosomal translocation wherein fusions occur between the EWS RNA Binding Protein 1 (*EWSR1*) gene and one of the ETS family genes (*FLI-1*), resulting in an aberrant transcription factor that reprograms gene expression. Current survival rates are around 70–80% for patients with standard-risk disease and ~30% for patients with metastatic disease. Survivors often experience important long-lasting adverse effects resulting from treatment, in addition to long-term consequences of the disease, such as limb amputations [16,17,18].

Changes in epigenetic programming during embryonic development are likely key events in the origin of childhood cancers, including NB and ES, and epigenetic-modulating compounds have been investigated as therapeutic agents for these diseases [19,20,21,22,23]. We have previously reported that higher *G9a/Ehmt2* transcription may predict poor prognosis with shorter overall survival (OS) in patients with the Sonic Hedgehog (SHH) molecular subgroup of medulloblastoma, which is the main type of pediatric malignant brain tumor, and that inhibiting G9a activity can impair medulloblastoma cell viability [24]. Here, we investigate *G9a* and *GLP* as possible biomarkers of prognosis and therapeutic targets in NB and ES.

## 2. Results

### 2.1. Transcriptional Levels of G9a/Ehmt2 and GLP/Ehmt1 on Survival Rates in Patients with NB

**Figure 1:**
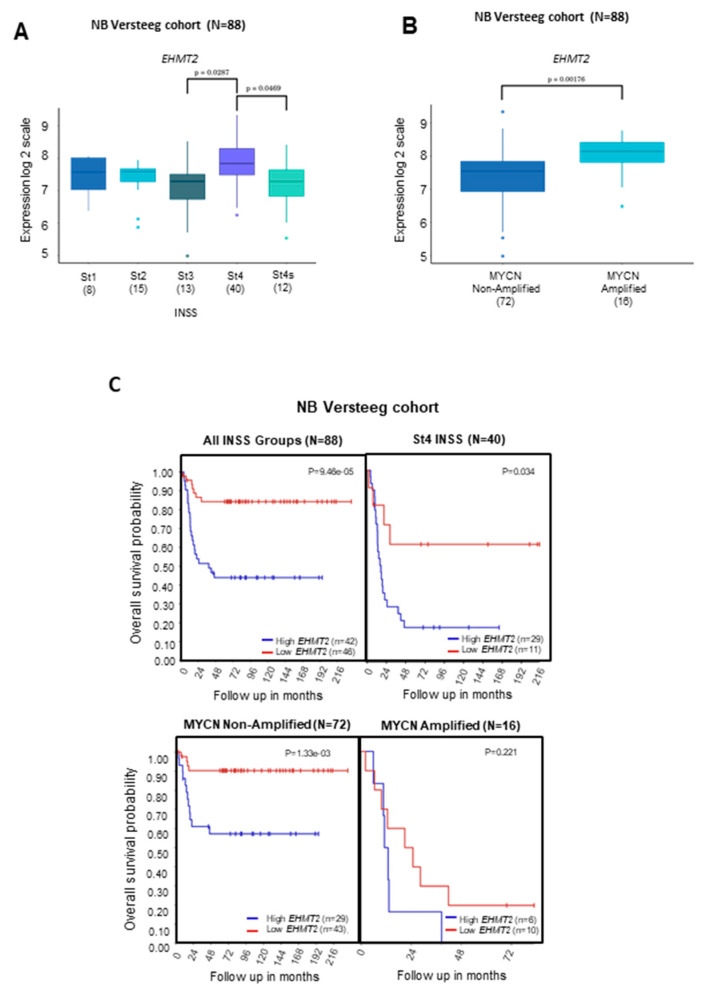
Transcript levels of *G9a* (*Ehmt2*) and OS of patients with NB tumors of different INSS stage and *MYCN* status. *G9a/Ehmt2* transcriptional levels were examined in transcriptome datasets comprising a total of 88 NB samples from patients [25] stratified by (**A**) INSS stage (St1, St2, St3, and St4) or (**B**) *MYCN* status (MYCN, *MYCN* single copy; MYCNA, *MYCN*-amplified). Samples were profiled on Affymetrix Human Genome U133 Plus 2.0 Array and normalized using the RMA method). Gene expression across all samples was normalized within the R2: Genomics Analysis and Visualization Platform (http://r2.amc.nl). All INSS groups and *MYCN* status (amplified, MYCNA; non-amplified, MYCN) in each dataset were compared by using a Kruskal–Wallis test for significance and Tukey’s tests for post hoc analysis using GraphPad Prism https://www.graphpad.com/features; *p* < 0.001 was considered to indicate statistically significant differences. (**C**) OS of NB patients carrying tumors with high and low levels of *G9a/Ehmt2* in all INSS stage groups and St4 INSS stage (upper panel); OS of NB patients carrying tumors with *MYCN*-non-amplified and *MYCN*-amplified (lower panel). OS was measured from the time of initial diagnosis to the date of death or the date of last follow-up using combined OS and gene expression data from all of the selected data sets. Survival distribution was estimated according to the Kaplan–Meier method using a median cut-off and log-rank statistics; *p* < 0.05 was considered statistically significant.

**Figure 2:**
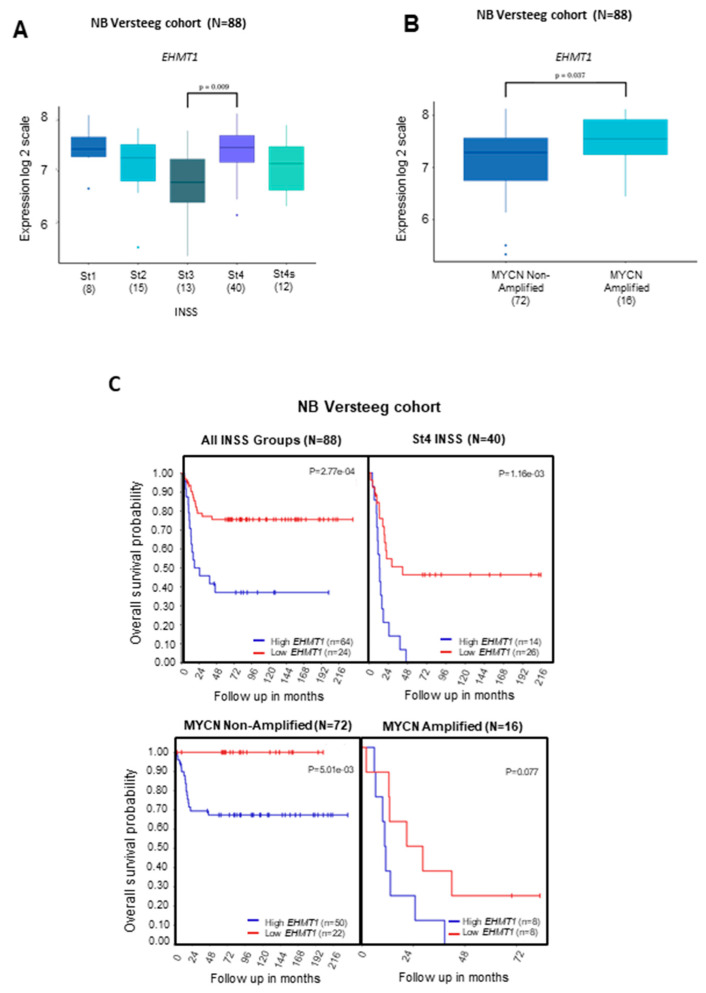
Transcript levels of *GLP* (*Ehmt1*) and OS in patients with NB tumors of different INSS stage and *MYCN* status. *GLP/Ehmt1* transcriptional levels were examined in transcriptome datasets comprising a total of 88 NB samples from patients [25] stratified by (**A**) INSS stage (St1, St2, St3, and St4) or (**B**) *MYCN* status (MYCN, *MYCN* single copy; MYCNA, *MYCN*-amplified). Samples were profiled on Affymetrix Human Genome U133 Plus 2.0 Array and normalized using the RMA method. Gene expression across all samples was normalized within the R2: Genomics Analysis and Visualization Platform (http://r2.amc.nl). All INSS groups and *MYCN* status (amplified, MYCNA; non-amplified, MYCN) in each dataset were compared by using a Kruskal–Wallis test for significance and Tukey’s tests for post hoc analysis using GraphPad Prism; *p* < 0.001 was considered to indicate statistically significant differences. (**C**) OS of NB patients carrying tumors with high and low levels of *GLP/Ehmt1* in all INSS stage groups and St4 INSS stage (upper panel); OS of NB patients carrying tumors with MYCN non-amplified and MYCN amplified (lower panel). OS was measured from the time of initial diagnosis to the date of death or the date of last follow up by using combined OS and gene expression data from all of the selected data sets. Survival distribution was estimated according to the Kaplan–Meier method by using a median cut-off and log-rank statistics; *p* < 0.05 was considered statistically significant.

Transcriptional analyses indicated that *G9a/Ehmt2* gene levels were higher in tumors belonging to the St4 International Neuroblastoma Staging System (INSS) stage compared to patients with St3 and St4s tumors, which can exhibit a better prognosis (Figure 1A). In addition, the *G9a/Ehmt2* transcriptional level was significantly higher in patients carrying *MYCN*-amplified tumors (Figure 1B). Patients with higher *G9a/Ehmt2* gene levels in NB tumors exhibited a significant reduction in OS (Figure 1C, upper panel). Moreover, NB patients with single copy *MYCN* and higher G9a transcriptional levels presented with a significant reduction in OS (Figure 1C). For *GLP/Ehmt1*, we found a significantly higher gene level in tumors belonging to the St4 INSS stage compared to patients with St3 (Figure 2A), as well as patients with *MYCN*-amplified tumors (Figure 2B). In addition, NB patients with higher *GLP/Ehmt1* gene levels in NB tumors with St4 INSS stage exhibited a significant reduction in OS (Figure 2C, upper panel). Also, patients carrying non-amplified MYCN tumors with higher *GLP/Ehmt1* gene levels exhibited a significant reduction in OS (Figure 2C, lower panel).

### 2.2. Transcriptional Levels of G9a/Ehmt2 and GLP/Ehmt1 on Survival Rates in Patients with ES

**Figure 3:**
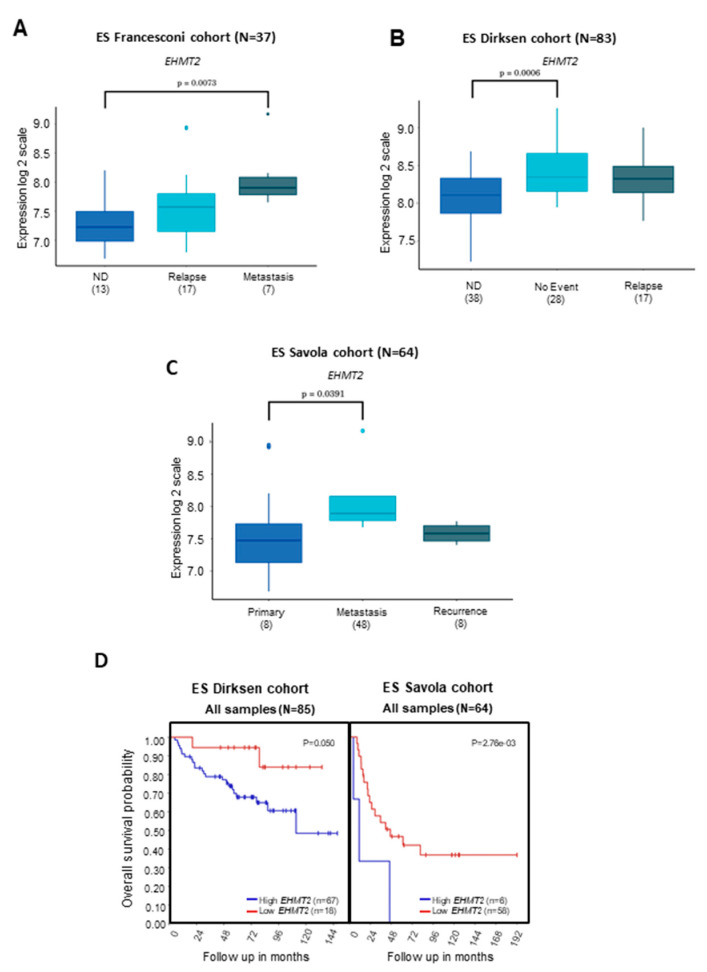
Transcript levels of *G9a* (*Ehmt2*) and OS in patients with ES tumors of different disease status. (**A**) *G9a/Ehmt2* transcriptional levels were examined in transcriptome datasets comprising a total of 37 ES tumor samples [26], and data were profiled according to the Affymetrix GeneChip Expression Analysis Technical Manual using HG-U133 Plus 2.0 Array. Analysis in (**B**) 83 [27] and (**C**) 64 ES samples [28] from patients from different cohorts. Gene expression of the markers across all samples was normalized within the R2: Genomics Analysis and Visualization Platform (http://r2.amc.nl); *p* < 0.001 was considered to indicate statistically significant differences. ND—no evidence of disease. (**D**) OS of ES patients carrying tumors with high and low levels of *G9a/Ehmt2*. OS was measured from the time of initial diagnosis to the date of death or the date of last follow-up using combined OS and gene expression data from all of the selected data sets. Survival distribution was estimated according to the Kaplan–Meier method using a median cut-off and log-rank statistics; *p* < 0.05 was considered statistically significant.

**Figure 4:**
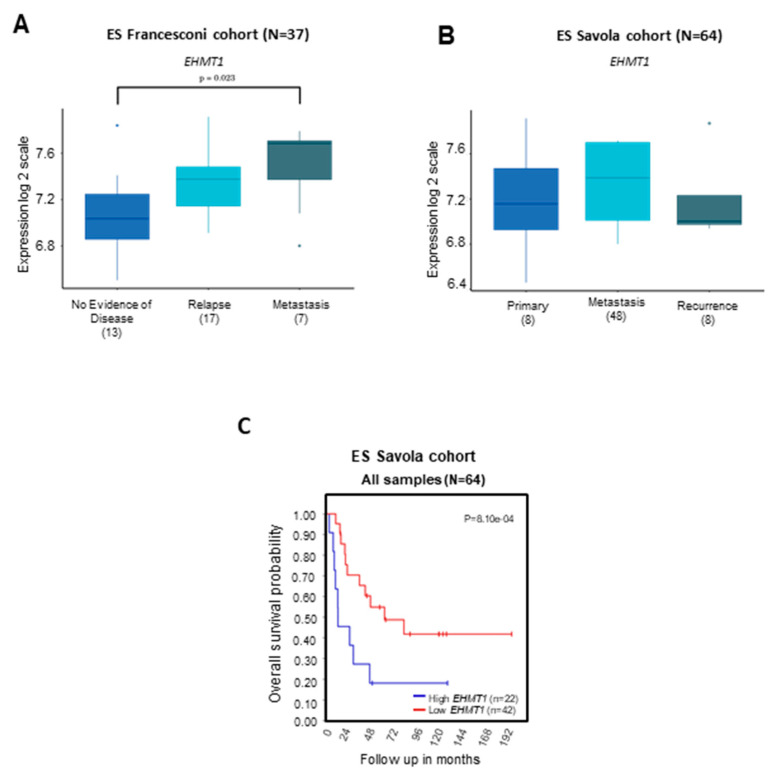
Transcript levels of *GLP* (*Ehmt1*) and OS in patients with ES tumors of different disease status. (**A**) *GLP/Ehmt1* transcriptional levels were examined in transcriptome datasets comprising a total of 37 ES tumor samples [26] and (**B**) 64 ES tumor samples from the Savola cohort [25]. Data were profiled according to the Affymetrix GeneChip Expression Analysis Technical Manual by using HG-U133 Plus 2.0 Array. Gene expression of the markers across all samples was normalized within the R2: Genomics Analysis and Visualization Platform (http://r2.amc.nl); *p* < 0.001 was considered to indicate statistically significant differences. ND—no evidence of disease. (**C**) OS of 64 ES patients from the Savola cohort [25] carrying tumors with high and low levels of *GLP* (*Ehmt1*). OS was measured from the time of initial diagnosis to the date of death or date of last follow-up using combined OS and gene expression data from all of the selected data sets. Survival distribution was estimated according to the Kaplan–Meier method using a median cut-off and log-rank statistics; *p* < 0.05 was considered statistically significant.

*G9a/Ehmt2* levels were higher in metastatic disease compared to patients with primary or relapsed disease (Figure 3A–C). Patients bearing ES tumors with higher *G9a/Ehmt2* levels showed a significant reduction in OS compared to patients with lower *G9a/Ehmt2* expression (Figure 3D). For *GLP/Ehmt1*, the gene level was higher in metastatic disease compared with patients with no evidence of disease (Figure 4A,B) In addition, patients bearing ES tumors with higher *GLP/Ehmt1* levels showed a significant reduction in OS compared with patients with lower *GLP/Ehmt1* levels (Figure 4C).

### 2.3. Correlation between Transcriptional Levels of G9a/Ehmt2 and GLP/Ehmt1 in Patients with NB

**Figure 5:**
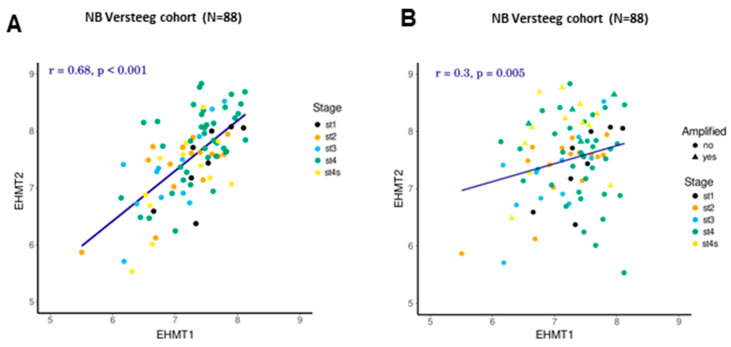
Correlation between G9a/Ehmt2 and GLP/Ehmt1 in NB tumors of different (**A**) INSS stages and (**B**) MYCN status. The straight line indicates the least squared error regression line. A correlation was calculated with Pearson’s product–moment correlation. Statistical significance was assessed with a paired t-test. Different colors indicate cancer stages. For the MYCN plot, a triangle shape indicates MYCN-amplified, and a circle shape indicates non-MYCN-amplified; r and *p* values are indicated.

To assess whether there is an association between transcriptional levels of G9a/Ehmt2 and GLP/Ehmt1 in NB, we calculated a Pearson’s product–moment correlation. There was a significant correlation between G9a/Ehmt2 and GLP/Ehmt1 levels in NB tumors of different stages (r = 0.68, *p* < 0.001; Figure 5A) and also when tumors were stratified by MYCN status (r = 0.3, *p* = 0.005; Figure 5B). There was no significant correlation between G9a/Ehmt2 and GLP/Ehmt1 expression in ES tumors.

### 2.4. G9a/GLP Inhibition Reduces NB and ES Cell Viability

**Figure 6:**
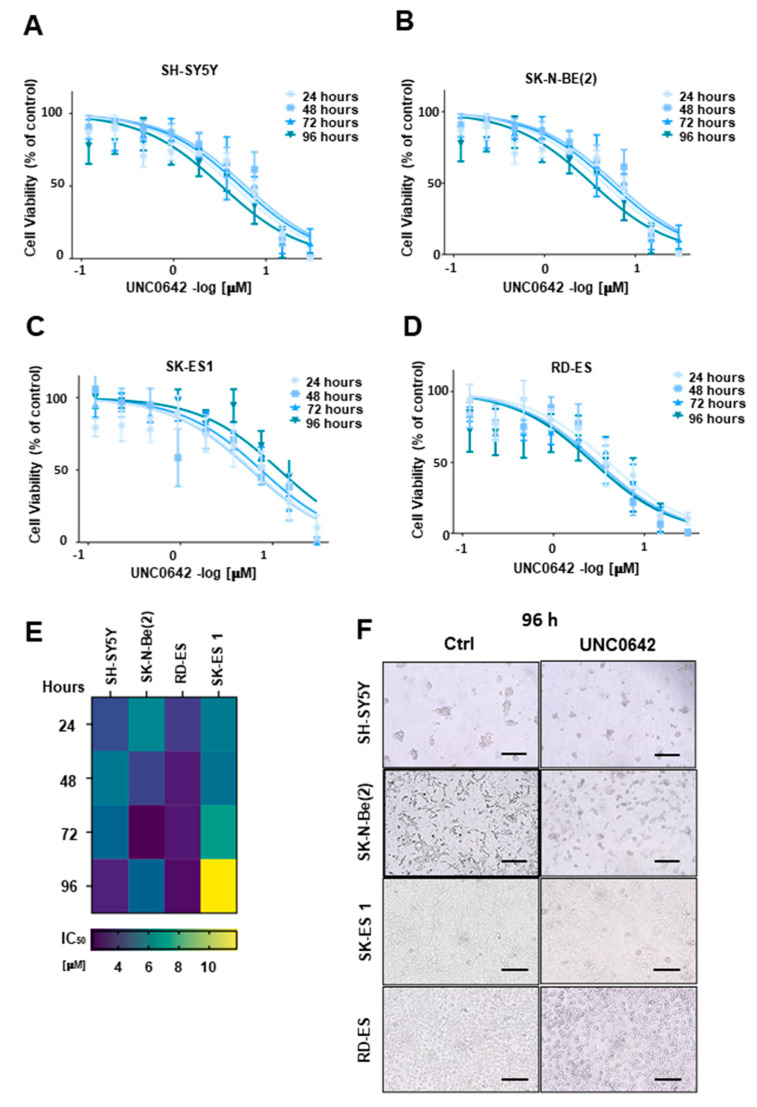
The G9a/GLP inhibitor UNC0642 reduces the viability of human NB and ES cells. Viability of (**A**) SH-SY5Y, (**B**) SK-N-Be(2), (**C**) SK-ES1, and (**D**) RD-ES human cells treated with 9 different concentrations (ranging from 0.1 to 30 μM) of UNC0642 for 24, 48, 72, or 96 h. Cell viability was assessed by using the Trypan blue exclusion method in a Neubauer chamber. All assays were performed in triplicate and repeated in three independent sets. For EC_50_ calculation, data were fitted in a dose response curve (GraphPad Prism) using the equation: *y = min* + (*max − min*)/(1 + 10^((*LogEC*50 − *x*)*Hillslope + *Log* ((*max* − *min*)/(50 − *min*) − 1))). (**E**) Heatmap showing EC_50_ (μM). The color key represents the average EC_50_ (μM) values: purple indicates low EC_50_, whereas yellow indicates high EC_50_. The EC_50_ doses ranged from 2.3 to 11.8 μM. The average EC_50_ was calculated based on the cell count of 3 biological replicates across NB and ES cell lines at 24, 48, 72, or 96 h using GraphPad Prism. (**F**) A representative image showing the effect of UNC0642 on cell viability reduction and altered cell morphology after treatment with 3.8 μM for NB SH-SY5Y and SK-N-Be(2) cells and ES RD-ES cells; and 7.5 μM for SK-ES1 cells at 96 h. Scale bars, 200 μm.

In order to verify whether the inhibition of G9a and GLP affects cell growth, we evaluated NB and ES cell viability after treatment with the G9a/GLP inhibitor UNC0642 for 24, 48, 72, or 96 h. The results showed that UNC0642 consistently and dose-dependently reduced cell viability in both SH-SY5Y (*MYCN* single copy) and SK-N-Be(2) (*MYCN*-amplified) NB cells, as well as SK-ES1 and RD-ES ES cells (Figure 6A–D). At 48, 72, and 96 h after G9a inhibition, we observed a lower EC50 dose in SK-N-Be(2) compared to SH-SY5Y NB cells, as well as lower EC50 values in RD-ES cells compared to SK-ES1 EB ES cells, indicating differential sensitivities (Figure 6E, Table 1). In addition, G9a/GLP inhibition induced a change in morphology in cell lines accompanied by the appearance of smaller NB neurosphere-like cells in SH-SY5Y cultures, as well as the appearance of short neurite-like extensions in ES cells (Figure 6F).

### 2.5. Expression of G9a/Ehmt2 and GLP/Ehmt1 mRNA Levels in NB, ES, and Non-Tumoral Human Cells

**Figure 7:**
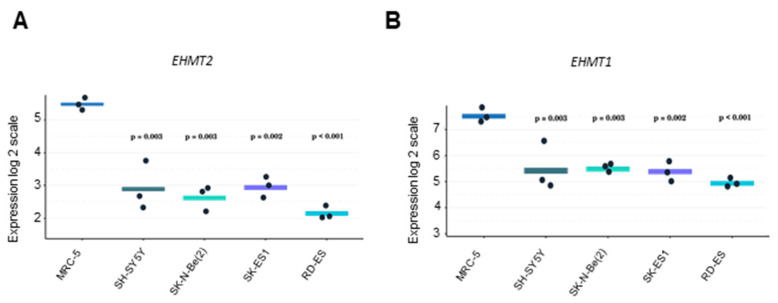
Expression of *G9a/Ehmt2* and *GLP/Ehmt1* in NB and ES cells, as well as human fibroblasts. (**A**) *G9a/Ehmt2* and (**B**) *GLP/Ehmt1* mRNA was assessed via qRT-PCR in the fibroblast line MRC-5, NB cell lines SH-SY5Y and SK-N-Be(2), and ES cell lines SK-ES1 and RD-ES. The mean Ct value for each gene was calculated relative to ACTB from experiments performed in triplicate. One-way ANOVA followed by Tukey’s post hoc tests was used; *p* values compared to control cells are indicated in the figure.

To identify transcriptional levels of *G9a/Ehmt2* and *GLP/Ehmt1* in NB and ES cells, we analyzed SH-SY5Y, SK-N-Be(2), SK-ES1, and RD-ES cells via qRT-PCR, in addition to analyzing human MRC-5 fibroblasts as a non-tumoral cell type. This analysis showed lower *GLP/Ehmt1* and *G9a/Ehmt2* levels in all tumor cell lines (SH-SY5Y, SK-N-Be(2), SK-ES1, and RD-ES) compared with MRC-5 human fibroblasts (Figure 7).

## 3. Discussion

This study demonstrated that *G9a/Ehmt2* and *GLP/Ehmt1* expression levels were higher in NB tumors of the St4 INSS stage and *MYCN*-amplified tumors, which are characteristic of a poorer prognosis. In ES, higher *G9a/Ehmt2* was found in metastatic disease compared to non-metastatic disease. In addition, both in NB and ES, higher *G9a/Ehmt2* and *GLP/Ehmt1* levels were significantly associated with shorter patient OS. Also, we found that patients with high *GLP/Ehmt1* levels carrying tumors with a non-amplified *MYCN* showed a significant reduction in OS survival compared with lower gene levels. Furthermore, there was a significant correlation between *G9a/Ehmt2* and *GLP/Ehmt1* transcript levels in NB.

It is well known that amplification of the *MYCN* gene is a biomarker for NB and it is highly associated with highly aggressive tumors and advanced stages of the disease. However, approximately 60% of high-risk NB tumors are non-*MYCN*-amplified [29,30,31]; therefore, these findings may explain this pattern of OS survival in patients with non-amplified tumors, which is consistent with our in silico analysis. Together, these findings indicate that high *G9a/Ehmt2* may be a marker of more severe disease. Finally, our cell culture experiments provide early-stage evidence suggesting that inhibiting G9a can lead to antitumor effects in both NB and ES. Consistent with previous findings [32], *MYCN*-amplified cells were more sensitive to G9a inhibition compared to a non-*MYCN*-amplified cell line. However, our results on G9a inhibition are limited to in vitro effects in cell lines and do not confirm the specific nature of the mechanism of action or the broader epigenetic and cellular effects; thus, further experiments are necessary to confirm and elaborate on this finding. We confirmed *G9a/Ehmt2* mRNA expression in all of the cell lines that were used in the study. Surprisingly, we observed a higher expression in the non-tumor cell model (MRC-5). This fibroblast line, which we used as a model of a non-tumoral embryonic cell, may not be an appropriate control for NB and ES non-tumoral cells of origin. MRC-5 fibroblasts are human fetal cells derived from embryonic lung tissue. The crucial role of G9a in fibroblast activation during lung metastasis [33,34], as well as the possibility of G9a upregulation during embryonic development [35,36], may possibly explain the high level of G9a mRNA that we observed in MRC-5 cells. An additional limitation is that we did not measure G9a protein levels to verify if they correlate with transcription activity.

Our results on *G9a/Ehmt2* expression and G9a inhibition largely replicate the results of previous studies [32,37]. Recently, García-Dominguez et al. found that *G9a/Ehmt2* expression correlates with poor prognosis assessed with OS and disease-free survival, as well as with metastasis occurrence, in patients with ES. A worsened prognosis is also associated with moderate-to-strong G9a protein content assessed via immunohistochemical analysis of tumors from a single institutional cohort of ES patients. Moreover, BIX01294 impairs proliferation and migration and stimulates autophagy in ES cells, and systemic treatment with BIX01294 reduced tumor growth and metastasis in a mouse model of ES [37]. Enrichment analysis identified *G9a/Ehmt2* as one of the identified main driver genes associated with chemotherapy resistance in ES [38]. The G9a protein content is higher in NB cell lines with *MYCN* amplification and in poorly differentiated or undifferentiated NB tumors, and G9a expression correlates with the expression of EZH2, which is a known oncoprotein in NB. In addition, G9a inhibition mediated by siRNA or small molecule inhibitors reduces cell growth in NB cell lines and selectively triggers apoptosis in *MYCN*-amplified NB cells. The effects of G9a inhibition may be associated with the reactivation of tumor suppressor genes [32]. Another study found that the specific G9a inhibitor BIX01294 impairs NB cell growth and proliferation, as well as NB tumorigenicity, in NOD/SCID mice. The antitumor effects were accompanied by features of autophagy, such as the appearance of membranous vacuoles and microtubule-associated protein light chain 3 (LC3B). Importantly, similar results were obtained in G9a-knockdown cells [39]. In addition to reducing NB cell proliferation and *MYCN* expression, BIX01294 inhibits cell mobility and invasion, induces apoptosis by stimulating caspase 8/caspase 3 activity, and modulates DNA methylation levels [40].

Some features of G9a may make it a particularly promising anticancer epigenetic target for NB and ES. For example, G9a overexpression in cancer may preferentially hinder the expression of tumor suppressors [41,42]; thus, G9a inhibition could be a strategy to restore tumor suppressor genes. Moreover, ES cells may be more sensitive to G9a inhibition compared to other tumor cells [37]. UNC0642 acts as a competitive substrate inhibitor, with improved pharmacokinetic properties being observed compared to those of first-generation G9a inhibitors, which make it suitable for in vivo testing [32,43]. Consistent with previous findings [32], a *MYCN*-amplified cell line had higher sensitivity to UNC0642 compared to a non-*MYCN*-amplified cell line. Therefore, the available evidence supports the further development of G9a and GLP inhibitors as candidate therapeutics for NB and ES.

## 4. Materials and Methods

### 4.1. Gene Expression

We conducted an analysis of *G9a/Ehmt2* and *GLP/Ehmt1* transcription using a previously described NB transcriptome data set consisting of 88 NB tumor samples from patients (Versteeg cohort). Original microarray data for these samples are available at the Gene Expression Omnibus (GEO) under accession number GSE16476 [25]. NB samples were profiled using the Affymetrix Human Genome U133 Plus 2.0 Array. NB tumors are classified by the INSS into 4 categories or stages (St1 to St4), which correspond to very low risk, low risk, intermediate risk, or high risk, respectively.

For ES, we analyzed three datasets, which included 37, 83, and 64 tumors obtained from patients [26,27,28]. Data for the ES samples from the Scotlandi et al. data set [26] were profiled using the Affymetrix Human Genome U133 Plus 2.0 Array. Original microarray data for these samples are available at the GEO under accession number GSE112102. For ES tumors from the Savola et al. data set [27], data were profiled using the Affymetrix Human Genome U133 Plus 2.0 Array, and original microarray data for these samples are available at the GEO under accession number GSE17679. Finally, we analyzed data from ES tumors from the Selvanathan et al. data set [28], which were profiled using the Affymetrix Human Exon 1.0 ST Array; original microarray data for these samples are available at the GEO under accession number GSE63157.

### 4.2. Cell Culture

Human NB cell lines SK-N-Be(2) (CRL-2271™) and SH-SY5Y (CRL-2266™), as well as human ES cell lines SK-ES1 (HTB-86™) and RD-ES (HTB-166™), were obtained from the American Type Culture Collection (ATCC, Rockville, MD, USA) and tested to confirm line identity and to rule out contamination. Cells were cultured in Eagle’s Minimum Essential Medium, F12 Medium, and RPMI-1640 Medium (Gibco, Grand Island, NE, USA) supplemented with 10% (*v*/*v*) fetal bovine serum (FBS, Gibco) and 1% (*v*/*v*) penicillin/streptomycin (Gibco). The culture medium was changed every 2–3 days. Cells were incubated in a humidified atmosphere of 5% CO_2_ at 37 °C. Human MRC-5 fibroblasts (ATCC CCL-171) were cultured in Dulbecco’s Modified Eagle Medium (Sigma-Aldrich, Saint Louis, MO, USA) supplemented with 10% (*v*/*v*) FBS (Gibco) and 1% penicillin/streptomycin (Gibco).

### 4.3. Cell Viability and Drug Treatment

Cells were seeded at 4000 to 5000 cells/well in 96-well plates, and, 24 h later, the cells were then treated with 9 different concentrations (ranging from 0.1–30 μM) of the G9a/GLP inhibitor UNC0642 [43] (Tocris, Bristol, UK) for 24, 48, 72, or 96 h. Dimethyl sulfoxide (DMSO, 100%) was used as a vehicle to dissolve UNC0642 and in the controls. To assess viability, the cell suspension was mixed with 0.4% trypan blue 1:1 and immediately counted in a hemocytometer.

### 4.4. Quantitative Reverse Transcription Polymerase Chain Reaction (qRT-PCR)

Total RNA was extracted using the ReliaPrep^TM^ RNA Cell Miniprep System (Promega, Madison, WI, USA) according to the manufacturer’s instructions and quantified in a NanoDrop (Thermo Fisher Scientific, Waltham, MA, USA). The cDNA was obtained using GoScript Reverse System (Promega, Madison, WI, USA) from 200 ng RNA, according to the manufacturer’s instructions. PowerUp SYBR Green Master Mix (Thermo Fisher Scientific) was used to quantify the mRNA expression levels of target genes in a QuantStudio 3 Real-time PCR System (Thermo Fisher Scientific), using gene-specific primers for *Ehmt1*, *G9a/Ehmt2*, and *ACTB*, which was used as an internal control (Table 2). Samples were analyzed in triplicate (n = 3).

### 4.5. Statistics

Gene expression levels across all of the samples were normalized using the RMA method within the R2: Genomics Analysis and Visualization Platform (http://r2.amc.nl) and presented in box plot format as log2-transformed signal intensity. Comparisons between the groups within each dataset were compared by using a Kruskal–Wallis test for significance and Tukey’s test for post hoc analysis via R and inscape software programs (version 4.1.2); *p* values of less than 0.05 were considered to indicate statistically significant differences. OS was measured from the time of initial diagnosis to the date of death or the date of last follow up using combined OS and gene expression data from all of the selected data sets. Survival distribution was estimated according to the Kaplan–Meier method using a median cut-off and log-rank statistics. For the association analysis, we performed the correlation calculated with Pearson’s product–moment correlation. Statistical significance was assessed with a paired *t*-test; *p* < 0.05 was considered to be statistically significant.

In cellular experiments, all of the assays were performed in triplicate and repeated in three independent sets. For calculation of EC_50_, data were fitted in a concentration–response curve (GraphPad Prism) by using the following equation: *y* = *min* + (*max* − *min*)/(1 + 10^((*LogEC*50 − *x*)*Hillslope + *Log* ((*max* − *min*)/(50 − *min*) − 1))). Data for qRT-PCR were analyzed using GraphPad Prism with one-way analysis of variance (ANOVA) followed by Tukey’s post hoc tests.

## 5. Conclusions

Our findings support a role for G9a and GLP in promoting NB and ES progression, suggesting that they are indicative of a worse disease prognosis. Also, our results, together with the results of previous studies, are consistent with the viewpoint that the pharmacological inhibition of G9a/GLP may be a novel promising adjunctive therapeutical strategy based on epigenetic regulation for the treatment of these pediatric solid tumors.

## Figures and Tables

**Table 1:** EC50 values (μM) of NB and ES cells treated with the G9a/GLP inhibitor UNC0642.

Time (h)	SH-SY5Y	SK-N-Be(2)	SK-ES 1	ED-ES
24	4.5 ± 0.08	4.5 ± 0.09	1.9 ± 0.07	2.6 ± 0.09
48	6.0 ± 0.07	4.2 ± 0.07	4.6 ± 0.06	2.3 ± 0.06
72	5.3 ± 0.06	2.4 ± 0.07	7.5 ± 0.07	2.0 ± 0.10
96	3.3 ± 0.08	2.0 ± 0.10	7.4 ± 0.08	2.6 ± 0.07

Values are means ± SEM of n = 3.

**Table 2:** Primers used for qRT-PCR measurement of gene expression.

Gene	Primers
*Ehmt1*	primer sense 5′-GCT GCT GGG AGA AGA GAC AC-3′ primer anti-sense 5′-AGC ATT TGC ATG ACT GCT GG-3′
*G9a/Ehmt2*	primer sense 5′-GAG GTC TAC TGC ATA GAT GCC-3′ primer anti-sense 5′-CAG ACG GTC CTG CTC CAG GGC-3′
*ACTB*	primer sense 5′-AAC TGG AAC GGT GAA GGT G-3′ primer anti-sense 5′-AGA GAA GTG GGG TGG CTT TT-3′

## Data Availability

The data presented in this study are available upon request.
